# Classification of Walking Environments Using Deep Learning Approach Based on Surface EMG Sensors Only

**DOI:** 10.3390/s21124204

**Published:** 2021-06-18

**Authors:** Pankwon Kim, Jinkyu Lee, Choongsoo S. Shin

**Affiliations:** Department of Mechanical Engineering, Sogang University, 35 Baekbeom-ro, Mapo-gu, Seoul 04107, Korea; kimpankwon@gmail.com (P.K.); jkl3921@gmail.com (J.L.)

**Keywords:** surface electromyography (sEMG), deep learning, non-handcrafted feature, walking environments, artificial neural network

## Abstract

Classification of terrain is a vital component in giving suitable control to a walking assistive device for the various walking conditions. Although surface electromyography (sEMG) signals have been combined with inputs from other sensors to detect walking intention, no study has yet classified walking environments using sEMG only. Therefore, the purpose of this study is to classify the current walking environment based on the entire sEMG profile gathered from selected muscles in the lower extremities. The muscle activations of selected muscles in the lower extremities were measured in 27 participants while they walked over flat-ground, upstairs, downstairs, uphill, and downhill. An artificial neural network (ANN) was employed to classify these walking environments using the entire sEMG profile recorded for all muscles during the stance phase. The result shows that the ANN was able to classify the current walking environment with high accuracy of 96.3% when using activation from all muscles. When muscle activation from flexor/extensor groups in the knee, ankle, and metatarsophalangeal joints were used individually to classify the environment, the triceps surae muscle activation showed the highest classification accuracy of 88.9%. In conclusion, a current walking environment was classified with high accuracy using an ANN based on only sEMG signals.

## 1. Introduction

Recent research in the field of robotic walking assistance exoskeletons or prostheses has seen many studies that look to achieve natural movement of the assisting robot through communication between the user and robot [1,2,3]. This human-robot interaction is intended to enable robots to recognize the user’s intended motion through cognitive interactions that occur between the human user and the robot that take place over various communication channels [4,5]. Intention recognition is important to accomplish synchronization between the motion of the robot and the human [6,7]. The other studies have reported various methods for recognizing the user’s intended movement based on bioelectrical signals, such as electromyography (EMG), electroencephalography (EEG), and electrooculogram (EOG) signals [8,9,10].

Surface electromyography (sEMG) signals contain neural information associated with human movement [3,11]. Human motion intention can be recognized by analyzing EMG signals, and that motion can also be classified to appropriately control any assisting devices [12,13,14]. However, analyzing sEMG signals is difficult due to their complicated patterns and non-linear nature [15]. Neural networks have the ability to understand and analyze complex systems, as such, in recent years they have been applied in many fields such as pattern recognition and adaptive control [5,16]. In particular, Morbidoni et al. used artificial neural networks (ANN) to classify the gait phase by applying the sEMG signals as input data [17]. Therefore, neural networks method and EMG signals can be effectively exploited to recognize the intended motion and to classify human motion.

Previously, both machine learning and traditional pattern recognition methods have utilized handcrafted features that were manually extracted from EMG signals in order to classify human movement [18,19]. However, the performance of these algorithms was greatly influenced by their handcrafted features, meaning their performance often depends on the experience of the engineer and the design of the feature extraction method [20,21]. Recently, it has been shown that extracting features using deep learning is more robust than relying on handcrafted features. For instance, Morbidoni et al. reported that a deep learning feature-based method was able to classify the gait phase with higher accuracy than a handcrafted features-based approach [17]. Roy et al. also proposed a deep learning-based classification framework that classifies hand motion with high accuracy [22]. In that study, the authors adopted an approach where features were extracted using deep learning as this leads to better overall classification performance.

Users of walking assistive devices will inevitably encounter various kinds of environments outside of flat, level ground during their daily life. Proper changes to the control of those walking assistive devices are required to adapt to the changes that occur in the sEMG signals and in the kinematics/kinetics of the joints in the lower extremities as they attempt to tackle various walking environments [23,24,25,26]. As such, classifying and/or recognizing the current walking environment is the first requirement in order to appropriately control the assistive device [27]. To the best of our knowledge, however, no study has attempted to classify the competing five conditions, including a flat-ground, upstairs, downstairs, uphill, and downhill walking, using only sEMG signals. sEMG signals have often been used in the classification of patterns as inputs to machine learning algorithms or artificial neural network (ANN); relying on these kinds of signals has been proven as a valid approach to classifying nonlinear data or complicated patterns [28]. Motivated by the state of research described above, the purpose of this study is to classify the current walking environment based on the entire sEMG profile from selected muscles in the lower extremities using an ANN.

## 2. Materials and Methods

### 2.1. Participants

Twenty-seven male students (age: 24.5 ± 2.7 years, height: 1.73 ± 0.04 m, mass: 69.0 ± 7.99 kg, BMI: 22.9 ± 2.2 kg/m^2^) participated in this study. Prior to participation, all participants were asked to sign an informed consent form approved by the Institutional Review Board (IRB); all participants were capable of ascending and descending stairs and slopes without any external assistance.

### 2.2. Experimental Protocol

**Figure 1:**
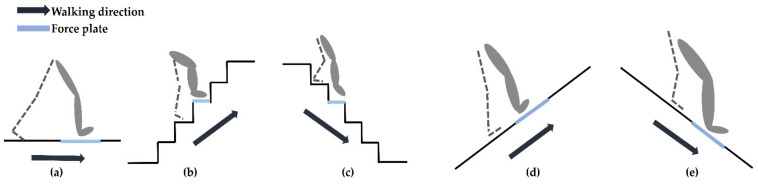
The five walking environments tested in this work (**a**) flat-ground, (**b**) upstairs, (**c**) downstairs, (**d**) uphill, and (**e**) downhill. A force plate was embedded in different positions according to each walking environment as shown by the blue line in each diagram. The force plate was embedded in the floor of the flat- ground environment as shown in (**a**). The experimental staircase was designed with five steps, here the force plate was embedded in the third step as shown in (**b**,**c**). The sloped walkway was made of three pieces joined together, the force plate was embedded in the second piece as shown in (**d**,**e**).

All participants walked barefoot in the following five environments: on flat-ground, upstairs, downstairs, uphill, and downhill (Figure 1). For the flat-ground environment, the participants walked along a straight and level 6 m walkway. For the stairs, the participants walked up and down a total of 5 steps (with each step 0.60 m in length, 0.25 m in width, 0.24 m in height) and there was a force platform embedded in the third step. For the uphill and downhill environments, a walkway with a 15° slope was used. The slope angle of 15° was selected, as it has been suggested in previous research that, at this angle, the effects of slope that come with walking uphill and downhill are apparent [29,30]. The sloped walkway consisted of three pieces, with each piece being 0.61 m in length and 0.76 m in width. The three pieces were joined together so that the participants could take a few natural steps along the walkway; a force plate was embedded below the second piece. Prior to the actual trials, each participant was instructed to perform several practice runs in the five experimental environments to become familiar with the procedures and instrumentation. During the tests, the participants were instructed to walk at a self-selected speed and to step on the force plate with their dominant leg each time. The dominant leg was defined as the more comfortable leg when kicking a ball [31,32]. All participants rested between each of the five walking tasks in order to prevent muscle fatigue. sEMG data from five successful trials was recorded for each environment, any trials where the participants did not correctly place their foot on the force plate were discarded.

### 2.3. Data Collection

**Figure 2:**
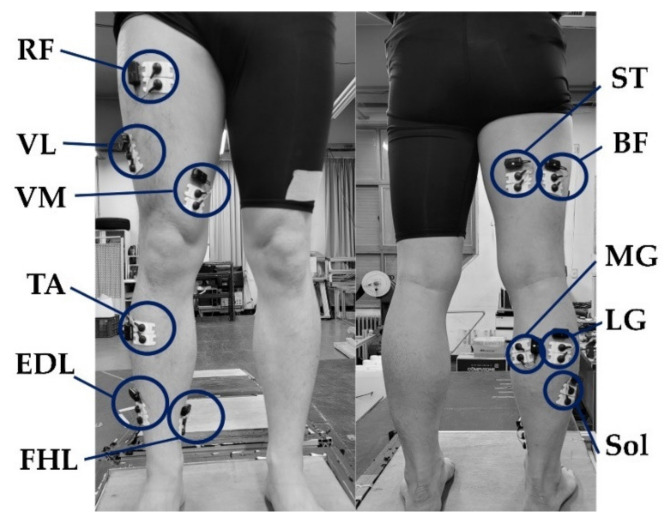
Attachment of electrodes. RF, VL, VM, ST, BF, MG, LG, Sol, TA, FHL, and EDL labels indicate the rectus femoris, vastus lateralis, vastus medialis, semitendinosus, biceps femoris, medial gastrocnemius, lateral gastrocnemius, soleus, tibialis anterior, flexor hallucis longus, and extensor digitorum longus, respectively.

A wireless EMG system (Wave plus wireless, Cometa, Milan, Italy) was used to record muscle activation data from the participants’ rectus femoris (RF), vastus medialis and lateralis (VM and VL), semitendinosus (ST), biceps femoris (BF), tibialis anterior (TA), soleus (Sol), medial and lateral gastrocnemius (MG and LG), flexor hallucis longus (FHL), and extensor digitorum longus (EDL) at a sampling rate of 1200 Hz while walking. The sEMG sensors were attached to the muscle bellies, while an inter-electrode distance of 20 mm at the recommended locations was maintained (Figure 2).

The force plate (9260AA6; Kistler, Winterthur, Switzerland) recorded data at a sampling rate of 1200 Hz, and their data collection was synchronized with that of the wireless EMG system to identify the stance phase in each participant’s walk. The force plate and EMG data for each walking environment were recorded simultaneously; in particular, the vertical ground reaction force (vGRF) was used to find the stance phase in each trial. The force plate was embedded in different positions as appropriate for each walking environment (Figure 1).

### 2.4. Data Processing

Muscle activation data was collected during the stance phase of each participant’s walk, this is defined as the period between the initial heel contact and toe-off. Initial heel contact was identified by finding the first frame in which the vGRF exceeded 20 N [33,34]. Toe-off was determined by the first frame after initial heel contact in which the vGRF returned 0 N.

**Figure 3:**
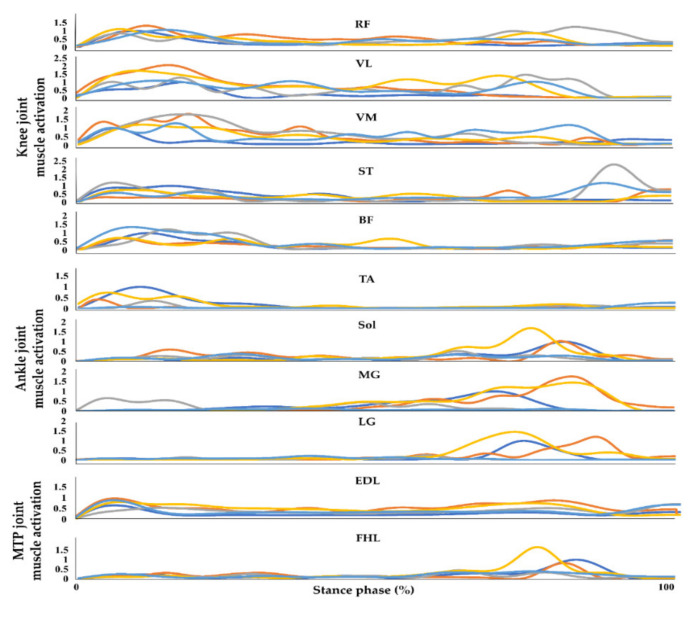
Example of an entire normalized sEMG profile from one trial of a subject collected during the stance phase while walking on flat-ground, upstairs, downstairs, uphill, and downhill. RF, VL, VM, ST, BF, TA, Sol, MG, LG, FHL, and EDL indicate the rectus femoris, vastus lateralis, vastus medialis, semitendinosus, biceps femoris, tibialis anterior, soleus, medial gastrocnemius, lateral gastrocnemius, flexor hallucis longus, and extensor digitorum longus, respectively. The blue, orange, grey, yellow, and sky-blue lines indicate walking on flat-ground, upstairs, downstairs, uphill, and downhill, respectively.

The muscle activation data from the selected muscles in the lower extremities were processed using MATLAB (MATLAB R2017b, Mathworks, Inc., Natik, MA, USA) [35]. The sEMG signals were processed to extract linear envelopes from the raw sEMG signals. Raw sEMG signals from walking on flat-ground, uphill, downhill, upstairs, and downstairs were passed through a fourth-order Butterworth filter for 20–500 Hz before being full-wave rectified. The rectified sEMG signals were subsequently passed through a fourth-order Butterworth low-pass filter at 10 Hz. The processed sEMG signals for all environments were normalized against each individual’s peak muscle activation amplitude during the flat-ground trial [36,37]. The individual peak muscle activation amplitude was defined as the maximum amplitude in the stance phase while walking on flat-ground. All sEMG signal data for each muscle during the stance phase were linearly interpolated to 1000 points to match the length of the input dataset before training and testing the model. An entire sEMG profile consists of the data collected during the stance phase from all muscles that were monitored. These data, from each trial of each individual, after being processed as described above, were used as the sole input to the ANN which then attempts to classify the walking environment (Figure 3). The number of input data points used for training and testing each model is shown in Table A1.

### 2.5. Walking Environment Classification

The processed sEMG signals from all walking environments were sorted by muscle, then the sorted sEMG signals were labeled to match the actual walking environment they were collected from (Table 1). Entire sEMG profiles of 135 successful trials for each walking environment obtained from 27 subjects during the stance phase were used as the sole input to the classification model. The input data was divided into 2 parts: 80% was used for training the classification model and 20% was used for testing it.

The ANN was used as a classifier. The processed sEMG muscle activation data were fed into the ANN as the input for the ANN to classify the walking environment. The ANN’s training model consisted of an input layer, a single hidden layer with the rectified linear unit (ReLU) activation function, and the output layers. Entire sEMG profiles from the muscles of each joint being monitored during the stance phase were fed to the input layer. The output layer then classified the input data as being from one of the five walking environments. A Softmax cross-entropy with logits was employed as a loss function, and Adaptive Moment Estimation (Adam) was used as an optimization algorithm to minimize the loss function [38,39,40,41].

**Figure 4:**
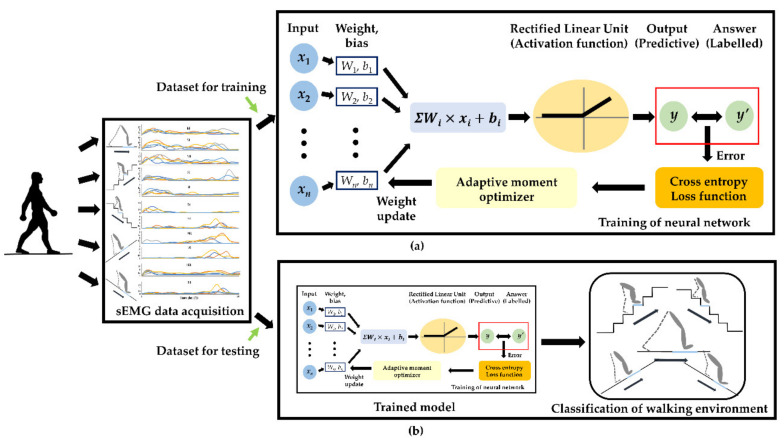
Schematics of the classification procedures using sEMG signals as the input to an artificial neural network (**a**) training the artificial neural network, (**b**) classification of walking environment.

Classification models were created for each muscle and combinations of muscles, each model’s accuracy was calculated after applying the test data. Specifically, each classification model was created by dividing the data from each joint’s flexor and extensor muscles (knee, ankle, and metatarsophalangeal (MTP) joint), and then the classification accuracy of each model was calculated after using the test dataset input (Figure 4).

Accuracy, sensitivity, and specificity are model evaluation indicators commonly used with classification problems [42]. For our ANN model, accuracy and confusion matrices were used to evaluate its classification performance. Accuracy of the ANN model is defined as an Equation (1).

(1) $$\mathrm{Accuracy}=\frac{N_{c}}{N_{total}}\times 100\%$$

where $N_{c}$ is the number of correctly classified environments, and $N_{total}$ is the total number of tests.

A confusion matrix is used to better quantify the specifics of the classification performance [43], this matrix is defined as follows:

$$A=\left[\begin{array}{ccc} a_{11} & \cdots & a_{1j}\\ \vdots & \ddots & \vdots \\ a_{i1} & \cdots & a_{ij} \end{array}\right]$$

where the elements of the matrix are defined by an Equation (2)

(2) $$a_{ij}=\frac{b_{ij}}{b_{total,i}}\times 100\%$$

where $b_{ij}$ is the number of samples for the ith walking terrain that are identified as the jth walking terrain, and $b_{total,i}$ is the total number of samples for the ith walking terrain. The diagonal elements in the matrix represent the percentage of correct classification events and are used to find the model’s accuracy, while the other elements in the matrix show the percentage of misclassified events.

In addition, sensitivity and specificity were calculated to further evaluate the model’s performance. They are defined as Equations (3) and (4).

(3) $$\mathrm{Sensitivity}=\frac{\mathrm{TP}}{\mathrm{TP}+\mathrm{FN}}$$

(4) $$\mathrm{Specificity}=\frac{\mathrm{TN}}{\mathrm{TN}+\mathrm{FP}}$$

where true positive (TP), true negative (TN), false positive (FP), and false negative (FN) are defined in Table 2.

In this study, the current walking environment was considered as positive; the other four walking environments were then considered negative for that particular trial. As such, five sensitivities and specificities were calculated, for when each walking environment was taken as the positive result.

## 3. Results

The ANN was able to classify each walking environment with a high degree of accuracy, achieving a success rate of 96.3% when using activation data from all the muscles being monitored (RF, VL, VM, ST, BF, MG, LG, Sol, TA, EDL, and FHL) (Table 3 and Table 4). The sensitivity and specificity for our model’s walking environment classification are shown in Table 4.

When separate flexor and extensor muscle group activations for each joint were used as the classifying parameters, data from MG, LG, and Sol, which are the ankle extensor muscles, achieved the highest classification accuracy (MG, LG, and Sol: 88.9%; ST and BF: 75.6%; VL, VM, and RF: 68.1%; FHL: 67.4%, TA: 63.0%; EDL: 45.2%; Table 5).

When individual muscle activation was used as the classifying parameter, the highest classification accuracy was obtained when using MG muscle activation data (MG: 81.5%, LG: 77.0%, ST: 72.6%, VM: 68.9%, Sol: 68.1%, RF: 67.4%, FHL: 67.4%, BF: 66.7%, TA: 63.0%, VL: 57.0%, and EDL: 45.2%; Table 6).

## 4. Discussion

This study proposed using an artificial neural network-based approach to classify whether a human user was walking on flat-ground, upstairs, downstairs, up a ramp, or down a ramp using only sEMG profiles collected from muscles in the lower extremities. When separating the flexor and extensor muscle groups of each joint (i.e., the knee, ankle, and MTP joints) to use as the input to the model, ankle extensors provided the best classification performance. This study proves it is possible to accurately classify the current walking environments based on an ANN using only sEMG signals. The results of this study show that classification accuracy was highest (96.3%) when using muscle activation data from all monitored muscles: the VM, VL, RF, ST, BF, TA, MG, LG, Sol, FHL, and EDL. It should be noted that this high accuracy is comparable and even higher than accuracies from the other studies that used a combination of multiple types of sensor. Kyeong et al. reported a classification accuracy of 96.1% when training and testing the model using the data obtained from multiple sensors (including sEMG, position sensors, GRF sensors, and interaction force sensors). However, the classification accuracy was 76.7% when training and testing the model using only the data obtained from the sEMG sensors [19]. Joshi et al. reported on their model, which classified the walking environment correctly 67.1% of the time when only relying on data from sEMG sensors [44]. In these studies, the walking environment was classified using a machine learning method based on time domain features calculated in the feature extraction process as a parameter. However, in our study, we utilized the entire sEMG profile collected during each participant’s stance phase as input data for our deep learning method. As muscle activation data from each time point may reflect any peak amplitude characteristics of the sEMG signals during the stance phase, we believe this is the reason for the higher accuracy of our model compared to other studies. Therefore, our results suggest that it is possible to classify the walking environment with a high degree of accuracy using only sEMG sensors when we use the entire muscle activation profile as input to the classification model.

This study found that muscle activation data from the ankle extensor group of muscles (i.e., the MG, LG, and Sol) gave the highest accuracy when classifying the walking environment. This result indicates that ankle extensor muscle activation provides the most important data when classifying the walking environment. This finding might be linked to differences in the lower limb joint kinetics of the sagittal plane when walking in different environments. Lay et al. reported that there were significant differences in the peak ankle joint moment in both early and late stance phases when walking on flat-ground, uphill, or downhill [45]. In addition, significant differences were found in the peak knee joint moment during the late stance phase between walking on flat-ground and downhill [45]. In the case of walking up and down stairs, the peak ankle joint moment has shown significant differences in the early stance phase between walking up stairs and walking down [26]. Differences in the peak knee joint moment appear in the late stance phase, regardless of whether we are walking upstairs or walking down, compared to walking on flat-ground [26]. Taking these previous findings together, we may conclude that the ankle joint has more significance than the knee joint in relation to classifying the current walking environment; this, in combination with the results of this study, suggests that muscle activation data from the ankle extensors should be monitored to properly control walking assistive devices as the user moves between different environments.

Although a high degree of accuracy while classifying the current walking environment was shown in this study, there is still room to increase the classification rate of our system for terrain detection, and to apply proper control of walking assistive devices. This study considered the current situation while walking in various environments, however, a system which could provide early detection of transitions between terrains would be preferable to enable timely control of walking assistive devices. Thus, future study into detecting transitions between the walking terrain is warranted. In addition, only male subjects were included in this study, so the current results cannot be generalized to females. To further enhance classification performance and generalize the classification model, more investigation into classifying various walking terrains through a larger sample size, including female subjects, is warranted.

## 5. Conclusions

This study proposed an ANN-based approach to classifying the user’s competing conditions as walking on flat-ground, upstairs, downstairs, uphill, or downhill. The main contribution of this study is to classify the walking environment by applying an entire sEMG profile from the stance phase as the only input to the ANN classification model. This study suggests that using all the sEMG data from every muscle group in the lower extremities is sufficient to determine a user’s gait characteristics as they change according to the walking conditions and that current deep learning methods can extract these gait characteristics successfully from these inputs. In conclusion, the current walking environment could be accurately classified using an ANN with only sEMG signals as input.

## Figures and Tables

**Table 1:** Labeling table. The walking environments are labelled in a sequence.

Walking Environment	Label
FGW	1
US	2
DS	3
UW	4
DW	5

FGW, US, DS, UW, and DW indicates walking on flat ground, upstairs, downstairs, uphill, and downhill, respectively.

**Table 2:** The definitions of TP, FP, FN, and TN.

	Prediction	Positive	Negative
Actual			
**Positive**		TP	FN
**Negative**		FP	TN

Abbreviations TP, FP, FN, and TN represent true positive, false positive, false negative, and true negative, respectively.

**Table 3:** Confusion matrix for walking environment classification using sEMG signals as input.

All Muscle Activation					
	FGW	US	DS	UW	DW
FGW	100	0	0	0	0
US	0	100	0	0	0
DS	0	0	96.3	0	3.7
UW	3.7	0	0	96.3	0
DW	0	3.7	3.7	3.7	88.9

FGW, US, DS, UW, and DW indicate walking on flat-ground, upstairs, downstairs, uphill, and downhill, respectively.

**Table 4:** The accuracy, sensitivity, and specificity of the model when using all muscle profiles.

	Walking Environment	All Muscle Activations
Accuracy (%)	All conditions	96.3
Sensitivity (%)	Flat-ground	100
	Upstairs	100
	Downstairs	96.3
	Uphill	96.3
	Downhill	88.9
Specificity (%)	Flat-ground	99.1
	Upstairs	99.1
	Downstairs	99.1
	Uphill	100
	Downhill	99.1

**Table 5:** The confusion matrix for classifying the walking environment using only sEMG signals from the flexor and extensor muscle groups of the ankle, knee, and metatarsophalangeal (MTP) joint.

Flexor							Extensor					
		FGW	US	DS	UW	DW		FGW	US	DS	UW	DW
Knee	FGW	74.1	3.7	0	14.8	7.4	FGW	77.8	3.7	0.0	3.7	14.8
	US	3.7	88.9	3.7	0	3.7	US	0.0	77.8	0.0	18.5	3.7
	DS	0	14.8	77.8	0	7.4	DS	7.4	0.0	74.1	3.7	14.8
	UW	18.5	0	3.7	74.1	3.7	UW	7.4	14.8	3.7	63.0	11.1
	DW	18.5	0	14.8	3.7	63	DW	11.1	18.5	14.8	7.4	48.1
Ankle	FGW	59.3	11.1	0.0	11.1	18.5	FGW	88.9	0.0	0.0	7.4	3.7
	US	3.7	48.1	29.6	3.7	14.8	US	7.4	92.6	0.0	0.0	0.0
	DS	0.0	3.7	81.5	3.7	11.1	DS	0.0	3.7	85.2	0.0	11.1
	UW	14.8	14.8	7.4	59.3	3.7	UW	11.1	0.0	0.0	88.9	0.0
	DW	7.4	11.1	7.4	7.4	66.7	DW	7.4	3.7	0.0	0.0	88.9
MTP	FGW	74.1	0.0	0.0	11.1	14.8	FGW	25.9	11.1	11.1	25.9	37.0
	US	3.7	81.5	7.4	0.0	7.4	US	7.4	51.9	29.6	11.1	0.0
	DS	11.1	7.4	55.6	7.4	18.5	DS	18.5	11.1	48.1	11.1	11.1
	UW	14.8	0.0	3.7	81.5	0.0	UW	22.2	11.1	25.9	40.7	0.0
	DW	22.2	7.4	18.5	7.4	44.4	DW	18.5	11.1	11.1	0.0	59.3

FGW, US, DS, UW, and DW indicate walking on flat-ground, upstairs, downstairs, uphill, and downhill, respectively. The knee flexor muscles are the vastus lateralis, vastus medialis, and rectus femoris. The knee extensor muscles are the semitendinosus and biceps femoris. The ankle flexor muscle is the tibialis anterior. The ankle extensor muscles are the medial/lateral gastrocnemius and soleus. The flexor and extensor muscles of the MTP joint are the flexor hallucis longus and extensor digitorum longus.

**Table 6:** The confusion matrix for classifying the walking environment using only sEMG signals from the individual muscles around the ankle, knee, and MTP joints.

Flexor								Extensor					
		FGW	US	DS	UW	DW			FGW	US	DS	UW	DW
RF	FGW	74.1	18.5	0.0	3.7	3.7	VL	FGW	66.7	7.4	7.4	14.8	3.7
	US	0.0	92.6	0.0	3.7	3.7		US	0.0	77.8	0.0	22.2	0.0
	DS	0.0	3.7	55.6	29.6	11.1		DS	3.7	0.0	55.6	7.4	33.3
	UW	3.7	11.1	7.4	55.6	22.2		UW	11.1	3.7	3.7	33.3	25.9
	DW	11.1	3.7	14.8	11.1	59.3		DW	14.8	3.7	11.1	18.5	51.9
VM	FGW	92.6	0.0	0.0	3.7	3.7	ST	FGW	74.1	0.0	0.0	7.4	18.5
	US	0.0	77.8	0.0	22.2	3.7		US	7.4	77.8	7.4	3.7	3.7
	DS	0.0	0.0	59.3	7.4	33.3		DS	0.0	22.2	66.7	0.0	11.1
	UW	11.1	14.8	0.0	59.3	14.8		UW	3.7	7.4	0.0	88.9	0.0
	DW	3.7	3.7	14.8	22.2	55.6		DW	29.6	7.4	3.7	3.7	55.6
BF	FGW	59.3	3.7	18.5	0.0	18.5	MG	FGW	85.2	0.0	0.0	11.1	3.7
	US	7.4	63.0	7.4	3.7	18.5		US	3.7	96.3	0.0	0.0	0.0
	DS	3.7	7.4	70.4	11.1	7.4		DS	0.0	0.0	63.0	0.0	37.0
	UW	7.4	3.7	3.7	85.2	0.0		UW	11.1	3.7	0.0	85.2	0.0
	DW	7.4	14.8	14.8	7.4	55.6		DW	14.8	0.0	7.4	0.0	77.8
LG	FGW	70.4	3.7	7.4	14.8	3.7	Sol	FGW	55.6	3.7	7.4	25.9	7.4
	US	7.4	88.9	0.0	0.0	3.7		US	7.4	85.2	3.7	3.7	0.0
	DS	7.4	0.0	66.7	3.7	22.2		DS	11.1	0.0	70.4	0.0	18.5
	UW	7.4	0.0	3.7	88.9	0.0		UW	14.8	3.7	3.7	74.1	3.7
	DW	0.0	0.0	25.9	3.7	70.4		DW	14.8	3.7	22.2	3.7	55.6
TA	FGW	59.3	11.1	0.0	11.1	18.5	FHL	FGW	74.1	0.0	0.0	11.1	14.8
	US	3.7	48.1	29.6	3.7	14.8		US	3.7	81.5	7.4	0.0	7.4
	DS	0.0	3.7	81.5	3.7	11.1		DS	11.1	7.4	55.6	7.4	18.5
	UW	14.8	14.8	7.4	59.3	3.7		UW	14.8	0.0	3.7	81.5	0.0
	DW	7.4	11.1	7.4	7.4	66.7		DW	22.2	7.4	18.5	7.4	44.4
EDL	FGW	25.9	11.1	11.1	14.8	37.0							
	US	7.4	51.9	29.6	11.1	0.0							
	DS	18.5	11.1	48.1	11.1	11.1							
	UW	22.2	11.1	25.9	40.7	0.0							
	DW	18.5	11.1	11.1	0.0	59.3							

FGW, US, DS, UW, and DW indicate walking on flat-ground, upstairs, downstairs, uphill, and downhill, respectively. RF, VL, VM, ST, BF, MG, LG, Sol, TA, FHL, and EDL indicate the rectus femoris, vastus lateralis, vastus medialis, semitendinosus, biceps femoris, medial gastrocnemius, lateral gastrocnemius, soleus, tibialis anterior, flexor hallucis longus, and extensor digitorum longus, respectively.

## Data Availability

Data sharing not applicable.

## Appendix A

**Table A1:** The number of input data points used for training and testing each model.

Muscle Used for Input	Number of Training Data Points	Number of Testing Data Points
All muscles	5,940,000	1,485,000
VL, VM, RF	1,620,000	405,000
ST, BF	1,080,000	270,000
LG, MG, Sol	1,620,000	405,000
FHL	540,000	135,000
EDL	540,000	135,000
RF	540,000	135,000
VL	540,000	135,000
VM	540,000	135,000
ST	540,000	135,000
BF	540,000	135,000
MG	540,000	135,000
LG	540,000	135,000
Sol	540,000	135,000
TA	540,000	135,000

RF, VL, VM, ST, BF, MG, LG, Sol, TA, FHL, and EDL indicate the rectus femoris, vastus lateralis, vastus medialis, semitendinosus, biceps femoris, medial gastrocnemius, lateral gastrocnemius, soleus, tibialis anterior, flexor hallucis longus, and extensor digitorum longus, respectively.
